# The role of exercise and nutrition in modulating inflammatory cytokines activity for obesity management

**DOI:** 10.3389/fendo.2025.1618360

**Published:** 2025-10-09

**Authors:** Sara Sobreviela Sánchez, Ravi Giusfredi Quevedo, Tiago Fernandes, Guilherme Wesley Peixoto da Fonseca

**Affiliations:** School of Physical Education and Sport, University of São Paulo, São Paulo, SP, Brazil

**Keywords:** exercise, nutrition, cytokines, inflammation, obesity

## Abstract

Obesity is recognized as a systemic disease characterized by chronic, low-grade inflammation. The persistent inflammatory state can be driven by secretion of cytokines from adipose tissue, also known as adipokines. In patients with obesity, adipose tissue releases elevated levels of pro-inflammatory cytokines that can have an autocrine and paracrine function. The main cytokines involved in this process include tumor necrosis factor alpha (TNF-α), interleukin-6, and interleukin-1 beta (IL-1β). These molecules actively contribute to metabolic dysregulation by interfering with insulin signaling pathways and facilitating the infiltration of immune cells into adipose tissue. Thus, a vicious cycle can be established in which inflammation perpetuates metabolic disturbances, increasing the risk of developing cardiovascular disease, type II diabetes mellitus, and other chronic conditions. On the other hand, physical exercise can release myokines with anti-inflammatory properties, such as interleukin-6 and irisin, which can positively modulate immune response. Regular physical activity and healthy eating patterns emerge as essential tools to counteract low-grade inflammation. A diet rich in bioactive compounds, such as antioxidants and polyunsaturated fatty acids, may also regulate cytokine expression, reinforcing the role of nutrition as a therapeutic strategy for obesity management. In conclusion, the role of inflammatory cytokines in obesity is central and managing their activity through non-pharmacological interventions, combining exercise and nutrition, represents a powerful tool to prevent long-term complications. However, more studies are needed to elucidate the exact molecular mechanisms by which nutrition and exercise modulate inflammation in obesity, in order to develop more effective interventions.

## Introduction

Adipose tissue was historically considered a passive reservoir for energy storage; however, it is now recognized as an active endocrine organ. Adipsin (1), one of the first adipokines discovered, was shown to modulate the immune system and energy metabolism, suggesting that the secretory capacity of adipose tissue could influence human health (2). The regulation and signaling of adipose tissue, as an endocrine organ, has recently gained significant attention for the management of obesity, a condition marked by chronic low-grade inflammation and altered adipokine secretion.

Obesity is a condition characterized by excessive accumulation of adipose tissue, affecting approximately 16% of the global adult population as of 2022, according to World Health Organization (WHO) data (3). The risk associated with obesity may be related to the function of adipose tissue via releasing pro-inflammatory cytokines. Moreover, secretion of several pro-inflammatory cytokines contributes to the development of obesity-associated comorbidities, such as insulin resistance, metabolic syndrome, cardiovascular diseases, and sarcopenia (4).

Regular physical activity has been proposed as an effective strategy to mitigate these adverse effects of obesity. Exercise training, beyond promoting weight loss, stimulates the release of myokines by skeletal muscle, such as interleukin-6 (IL-6), which engage in bidirectional crosstalk with adipose tissue and other organs modulating systemic inflammation (5, 6). Thus, physical exercise is an important tool to effectively manage obesity in clinical practice.

On the other hand, several nutrients, when incorporated into the diet, have been shown to modulate inflammation, including omega-3 fatty acids, polyphenols, and dietary fiber, which modulate immune responses through gut microbiota and inflammatory pathways (7). Dietary patterns can also provide health benefits, as reported with the Mediterranean diet (8). Furthermore, these benefits can be particularly important for obesity management, as obese patients may present elevated levels of inflammation, increasing the risk of metabolic diseases (9). However, few studies integrate exercise and nutritional interventions to modulate inflammatory cytokines activity.

Therefore, this narrative review aims to examine the interplay between cytokines, physical exercise, and nutrition in the context of obesity, with a focus on their mechanistic roles in modulating inflammation and their practical implications for clinical management. For this purpose, a comprehensive literature search was conducted using PubMed, covering publications from 1995 to 2025.

## Obesity and inflammation

Obesity is defined as the excessive accumulation of adipose tissue, identified in patients with a body mass index (BMI) equal or higher than 30 kg/m^2^ (10). Although BMI can be an easy measurement to acquire in clinical practice using only body weight and height, BMI lacks sensitivity for detecting excess adiposity, particularly in individuals with high muscle mass (11). However, despite normal BMI, patients may have excessive adipose tissue, a phenomenon known as normal weight obesity, and yet present an increased risk of obesity-related comorbidities (12). Thus, assessing body composition, especially percentage of fat mass, can assist in analyzing health risks related to obesity. Moreover, obesity is not always accompanied by other comorbidities. Preclinical obesity refers to an early stage of obesity where mild metabolic disturbance can occur, whereas clinical obesity can be related to organ dysfunction (13).

Adipose tissue is an active endocrine organ that secretes adipokine, cytokines and hormone-like substances involved in metabolic and inflammatory regulation (14). The transition from lean to obese is accompanied by a chronic low-grade inflammation and immune dysregulation, as well as enhanced release of pro-inflammatory cytokines, which can consequently promote obesity-induced comorbidities. Moreover, pro-inflammatory cytokines can contribute to skeletal muscle disorders, such as sarcopenia (15), presenting similar elevated inflammatory markers as obesity, like interleukin-1β (IL-1β), IL-6 and tumor necrosis factor alpha (TNF-α) (16). Obesity can also lead to the infiltration of immune cells, primarily macrophages, into skeletal muscle, reducing muscle density and impairing muscle function (17).

Regulation of gut microbiota has been proposed as an additional mechanism through which obesity can promote low-grade inflammation (18). The microbiota of patients with obesity seems to exhibit specific characteristics related to impaired diversity and microbial composition, differing from individuals with normal weight (19). In addition, there may be differences in the microbiota depending on associated-comorbidities in patients with obesity, suggesting an interplay between metabolic disorders and obesity meditated by gut microbiota (20). It appears that the microbiota of obese patients may provide more energy to the host, possibly through enhanced fermentation of dietary substrates and altered production of short-chain fatty acids (SCFAs), which influence energy balance and appetite regulation (21).

Low-grade inflammation has been widely associated with disruptions in glucose metabolism, commonly reported in patients with obesity and type II diabetes mellitus (22). Additionally, inflammation can increase the risk of cardiovascular diseases, especially when other risk factors coexist (23). Therefore, reducing low-grade inflammation caused by excessive adipose tissue accumulation can be a strategy to manage obesity. In this context, specific pro-inflammatory cytokines such as TNF-α, IL-1β, and IL-6 have emerged as key players in the pathophysiology of obesity and its complications.

## Tumor necrosis factor alpha

Tumor necrosis factor alpha (TNF-α), a pro-inflammatory cytokine secreted primarily by macrophages, has been associated with the pathogenesis of autoimmune diseases (24). TNF-α plays a central role in mediating inflammation by promoting apoptosis of damaged cells and increasing oxidative stress, contributing to metabolic dysfunction when chronically elevated (25). Glucose metabolism is also impaired with elevated TNF-α causing insulin resistance (26). Increased level of TNF-α has shown to impair intracellular insulin receptor signaling in adipocytes, hepatocytes, and skeletal muscle cells by inhibiting insulin receptor substrate-1 (IRS-1) (27). Additionally, it has been reported that the administration of TNF-α in human adipocytes reduces mRNA expression of glucose transporter type 4 (GLUT-4) (28). GLUT-4, a member of the glucose transporter family, translocates to the cell membrane and allows influx of monosaccharide into the cell cytoplasm, showing that the regulation of insulin signaling pathway can be mediated by inflammation promoted by high levels of adipose tissue (29).

Excessive release of TNF-α can also affect lipid metabolism. Patients with dyslipidemia have shown to have higher levels of this cytokine, which is associated with increased concentrations of triglycerides (TG) and low-density lipoprotein (LDL) (30). TNF-α can also inhibit the activity of lipoprotein lipase (LPL) in adipose tissue, an enzyme responsible for hydrolysis of triacylglycerol present in chylomicrons and very low-density lipoprotein (VLDL) (28). Although anti-TNF-α antibody therapy may play a role in obesity-related comorbidities, the inflammatory response observed in obesity is more likely a consequence of excess adiposity rather than its primary cause (31). On the other hand, if TNF-α inhibitors may cause an increase in body weight by impairing lipolysis, blocking TNF-α signaling could become a new strategy to treat skeletal muscle disorders with severely reduced weight loss, such as cancer cachexia (32).

Elevated TNF-α levels have been also associated with endothelial dysfunction and increased arterial stiffness, both key contributors to hypertension and atherosclerosis (33). Endothelin-1 can be involved in vasoconstriction of vascular smooth muscle cells, reducing the lumen of blood vessels that may lead chronically to persistent increases in blood pressure. These changes in vascular response through elevated secretion of TNF-α may become more severe with aging (34).

Exercise has shown to improve TNF-α levels in mice in a high-fat diet, suggesting that exercise may be a strong regulators of TNF-α (35). Several types of exercise have shown to modulate TNF-α levels, such as light-intensity walking (36), even though other studies have not demonstrated significant impact of exercise on reducing TNF-α (37, 38). These conflicting results may be related to the maintenance or transitory increase in pro-inflammatory cytokines in response to muscle damage caused by intense and prolonged bout of exercise (39, 40).

Nutritional strategies also play a crucial role in modulating TNF-α. The consumption of natural soy products seems to have positive effects on reducing plasma levels of TNF-α (41), which may be due to the effect of isoflavones as antioxidants (42). The combination of omega-3 fatty acids with curcumin supplementation has shown to reduce TNF-α expression and serum levels in patients with migraines (43). In patients with sarcopenia, protein supplementation, whether soy or whey protein, was also capable of decreasing serum TNF-α (**Table 1**) (44). Moreover, other compounds, such as coenzyme Q10 (45), or tannins (46), also seem to have an anti-inflammatory effect and significantly reduce TNF-α levels, due to its antioxidant capacity.

**Table 1:** Effect of nutritional interventions on inflammatory profile and obesity-related comorbidities.

Nutritional intervention	Cytokine modified	Outcomes
Omega-3 fatty acids (fish oil, flaxseed oil, DHA, and n-3 PUFA)	TNF-α, IL-1, IL-1β, and IL-6	Reduces serum levels and expression of TNF-α and IL-1 in patients with type II diabetes mellitus and coronary artery disease. Inversely correlated with plasma IL-6. Improves wall shear stress, left ventricular function, and blood pressure in pre-clinical studies.
Antioxidant–rich compounds (natural soy products, curcumin, coenzyme Q10, tannins, vitamin C and E)	TNF-α and IL-6	Reduces plasma levels of TNF-α. Reduces plasma and muscle-derived IL-6 after moderate exercise.
Protein supplementation (soy or whey protein)	TNF-α, IL-1β, and IL-6	Decreases serum TNF-α in patients with sarcopenia. Useful for reducing plasma IL-6 levels after moderate exercise. Reduces levels of TNF-α, IL-1β, and IL-6.
Vitamin D supplementation	TNF-α and IL-1β	Decreases serum levels of IL-1β and TNF-α in patients with type II diabetes mellitus.
Gut microbiota modulators (prebiotics and probiotics)	TNF-α and IL-6	May improve gut microbiota, reducing both systemic and intestinal inflammation.
Mediterranean diet	TNF-α, IL-1, IL-1β, and IL-6	Suppresses pro-inflammatory cytokines. Associated with improved biomarkers related to obesity comorbidities. Reduces low-grade inflammation.

**Figure 1:**
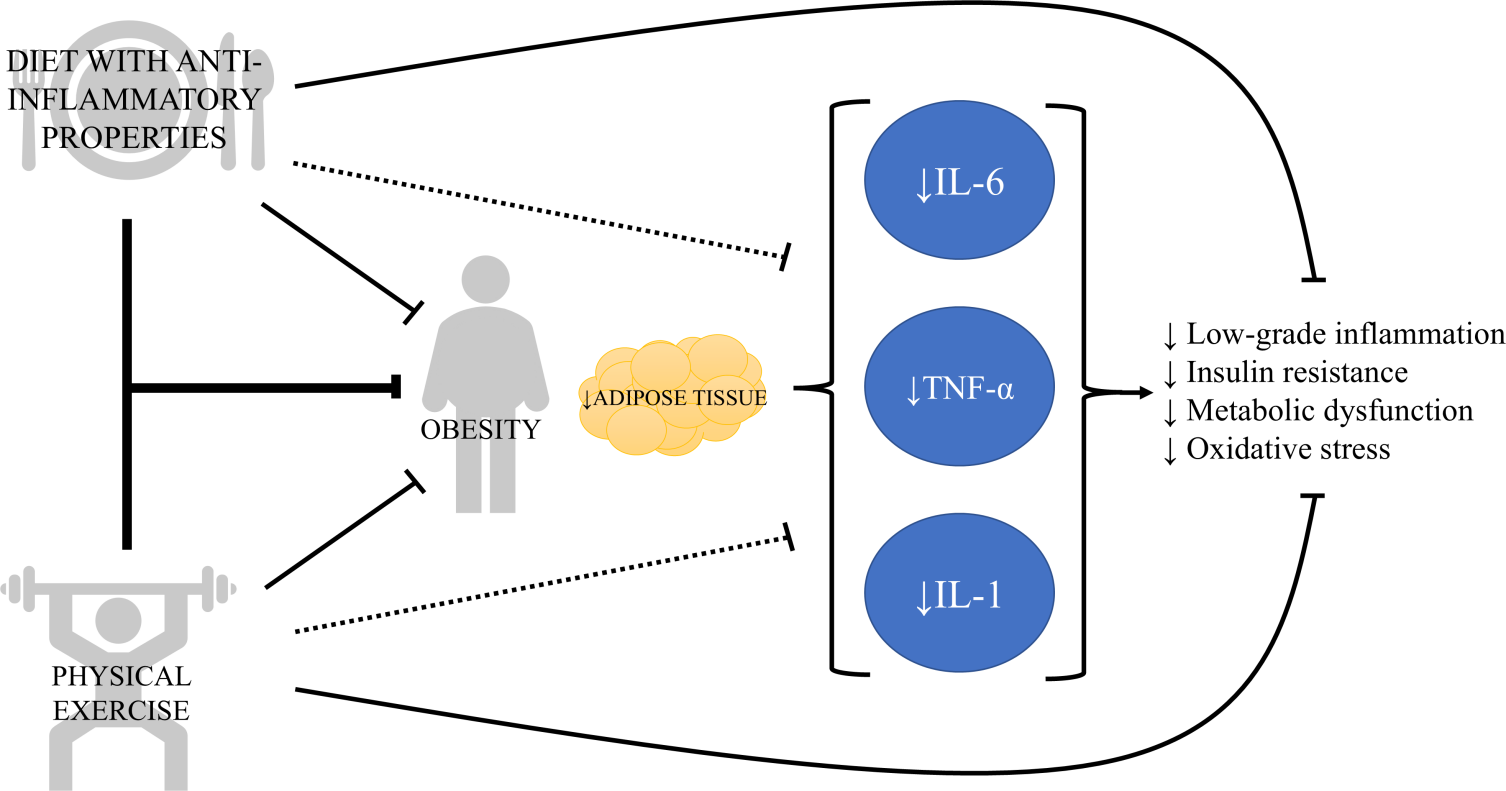
The vicious cycle of chronic inflammation in obesity and its modulation by lifestyle strategies. This diagram illustrates that obesity increases adipose tissue, which secretes pro-inflammatory cytokines such as interleukin-6 (IL-6), tumor necrosis factor alpha (TNF-α), and interleukin-1 (IL-1). These cytokines, in turn, contribute to insulin resistance, metabolic dysfunction, oxidative stress, and tissue damage, which physical exercise and diet with anti-inflammatory components can work synergistically or independently to counteract the chronic inflammation and obesity-related complications.

In summary, TNF-α is a pro-inflammatory cytokine secreted by macrophages in a healthy environment, but it can also be secreted by adipose tissue in obesity, showing strong association with health issues such as insulin resistance, dyslipidemia, sarcopenia and cardiovascular conditions. Low-intensity exercise and several bioactive compounds, including omega-3 fatty acids and antioxidants, can modulate inflammation through TNF-α (**Figure 1**). However, more studies are necessary to determine the impact of different training variables (i.e., intensity and volume), as well as the combination of exercise with nutritional strategies, on modulating TNF-α in patients with obesity.

## Interleukin-1

The interleukin-1 family comprises 11 cytokines that play a central role in regulating immune and inflammatory responses. Interleukin-1 (IL-1) is a pro-inflammatory cytokine essential for regulating immune response against infections. IL-1α and IL-1β are the most studied members as they possess strong pro-inflammatory effects. IL-1 works via innate immune response activating macrophages and neutrophils, while can enhance activation of T and B cells in adaptive immune response (47). Moreover, IL-1 induces local histamine release from mast cells, stimulating early vasodilation and increasing vascular permeability (48). IL-1 has shown to act synergistically with TNF-α through promoting cell death (49). As TNF-α, IL-1 can also be secreted by adipose tissue under pathological conditions.

Glucose metabolism is also affected by IL-1. It has been reported that blocking IL-1 can preserve pancreatic cell mass and function, while IL-1β can promote apoptosis of pancreatic beta cells in hyperglycemic conditions (50). The risk of atherosclerosis may also be increased in the presence of IL-1, since IL-1 is involved in vascular wall inflammation, via activation of monocytes and expression of adhesion molecules (51). Additionally, IL-1 in adipose tissue can promote macrophage infiltration and release of other pro-inflammatory cytokines, which creates a vicious cycle in the progression of obesity (52).

Interleukin-18 (IL-18), another pro-inflammatory cytokine belonging to the IL-1 family, can be considered essential in the inflammatory state of patients with obesity. Plasma levels of IL-18 are positively correlated with insulin resistance and may be related to the development of type II diabetes mellitus (53). Moreover, the contribution of IL-18 to chronic inflammation can be associated with facilitating formation of atherosclerotic plaques and endothelial dysfunction that can promote the development of cardiovascular diseases (54).

Interestingly, physical exercise can be a key strategy when it comes to reducing IL-1 family cytokine levels (**Figure 1**). It has been shown that physical exercise reduces IL-1β gene expression in patients with heart failure (55), which in turn may improve exercise capacity of these patients (56). In experimental studies with diabetic rats, swimming significantly reduced the expression of inflammatory cytokines, such as IL-1β (57). However, as of TNF-α, plasma levels of IL-1 may increase after intense exercise as a physiological response to strenuous muscle effort (58), making it difficult to assess the isolated impact of physical activity on IL-1.

Vitamin D and calcium supplementation through a drinkable yogurt has been associated with a decrease in serum levels of IL-1β and TNF-α in patients with type II diabetes mellitus (59). In addition, when supplemented only calcium no significant effects on inflammation were reported, suggesting that the anti-inflammatory effect may be specific to vitamin D (**Table 1**). Likewise, supplementation with flaxseed oil, a source of linolenic acid, significantly improved the expression levels of IL-1 and TNF-α in patients with diabetes and coronary artery disease (**Table 1**) (60). Dietary intake of docosahexaenoic acid (DHA) in mice has been shown to reduce wall shear stress, improve left ventricular function, and lower blood pressure compared to controls, suggesting that reduced local IL-1β expression can mediate these adaptations (61). These last two studies reaffirm the anti-inflammatory capabilities of omega-3 fatty acids and their benefits on obesity-related comorbidities.

The IL-1 family plays a critical role in regulating both innate and adaptive immune responses. Despite triggering low-grade inflammation, IL-1 has been linked to obesity-related comorbidities such as atherosclerosis and endothelial dysfunction. Supplementation of omega-3 fatty acids stands again as clinical strategy to modulate inflammation, while low-intensity exercise can modulate several isoforms of IL-1. Nonetheless, more studies are needed to understand the role of IL-1 family in the context of exercise and nutritional interventions in patients with obesity.

## Interleukin-6

**Figure 2:**
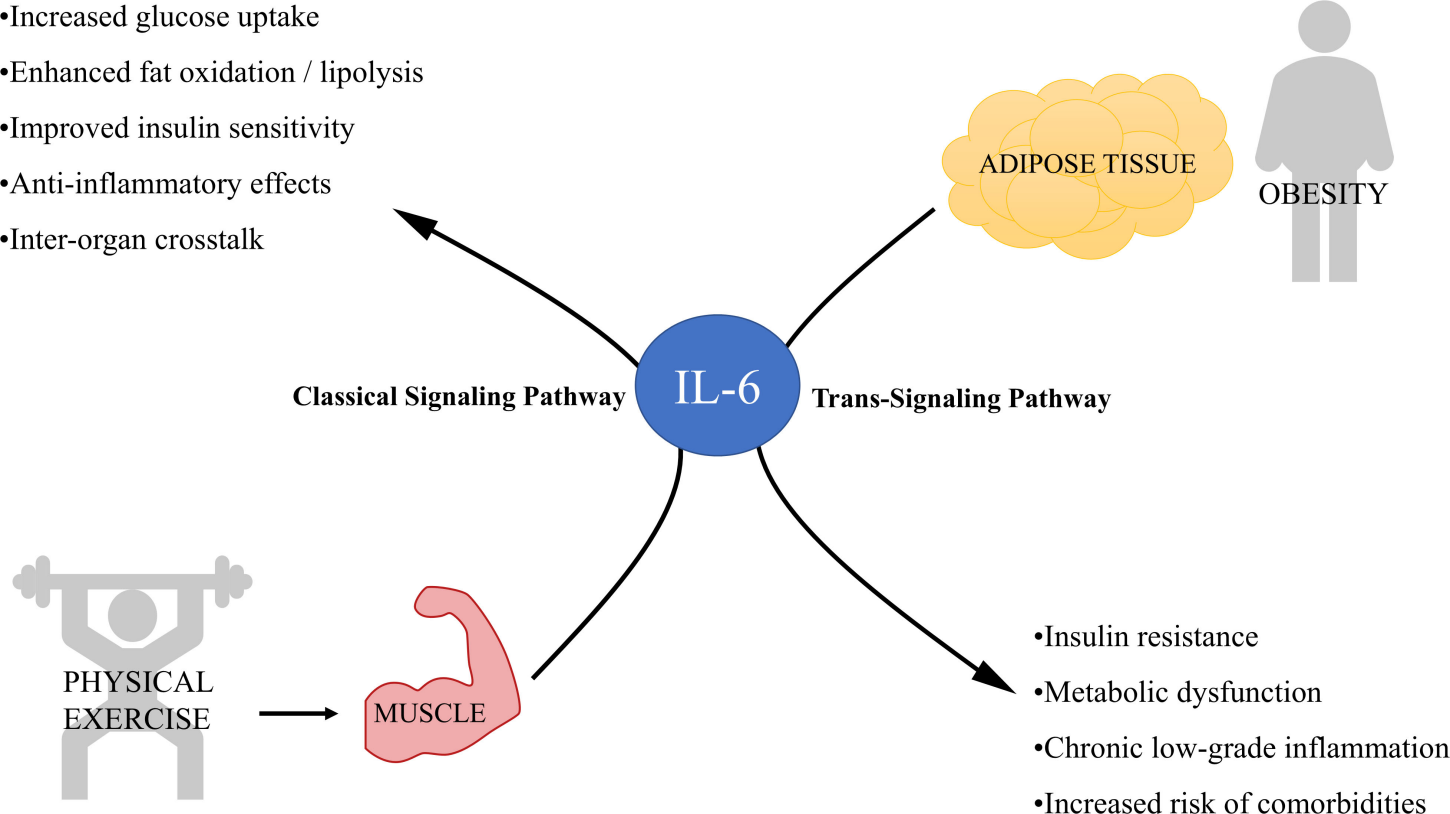
Dual role of interleukin-6 (IL-6) released from skeletal muscle and adipose tissue in metabolism and inflammation. This diagram illustrates the contrasting effects of IL-6 depending on its source and signaling pathway involved. On the left, physical exercise stimulates skeletal muscle to release IL-6, which primarily signals through the classical signaling pathway, producing anti-inflammatory effects. On the right, obesity leads to increased IL-6 secretion from adipose tissue, predominantly activating the trans-signaling pathway that is associated with chronic inflammation and metabolic dysregulation.

Interleukin-6 (IL-6) is a cytokine with both pro-inflammatory and anti-inflammatory properties (**Figure 2**) (62). Under normal conditions, IL-6 is required not only for inflammatory processes, but also for hematopoiesis, bone metabolism and coagulation (63), as well as for facilitating glycemic control and providing cross-talk, linking tissues such as intestinal L cells and pancreatic islets (5).

Mechanistically, two main signaling pathways can be activated by IL-6. In the classical signaling pathway, IL-6 binds to its membrane receptor (IL-6R), followed by dimerization of glycoprotein 130 (gp130) and activation of the JAK/STAT, MAPK and PI3K/AKT pathways (64). Through the trans-signaling pathway, IL-6 can also bind to molecules of the soluble IL-6 receptor (sIL-6R), which are generated through ectodomain release by metalloproteases (ADAM-10 and ADAM-17) (65). The expression levels of IL-6 receptor subunits vary among cell types and determine whether IL-6 classical signaling pathway, which primarily induces anti-inflammatory responses, or IL-6 trans-signaling pathway, generally associated with pro-inflammatory responses, is triggered (66). Moreover, the presence of other cytokines may modulate IL-6 response (67).

As mentioned before, low-grade inflammation in obese patients impacts negatively in insulin signaling pathways and causes insulin resistance, with IL-6 playing an important role in this process (68). In pre-clinical studies, IL-6 knockout mice developed obesity, associated with a disturbed carbohydrate and lipid metabolism (69). Indeed, these data were subsequently supported indicating increased body weight, impaired glucose tolerance and exacerbated insulin resistance in IL-6 knockout mice (70). In regards to lipid metabolism, IL-6 has been linked to the development of dyslipidemia and a massive mobilization of fatty acids that may lead to lipid accumulation in the myocardium, potentially causing cardiac lipotoxicity (71). In addition, IL-6 has been associated with increase risk of several types of cancer, including breast, liver, and colon cancer (72).

However, IL-6 can be somewhat paradoxical, as both harmful and beneficial effects have been reported. For instance, muscle-derived IL-6 has beneficial effects unlike that secreted during inflammation, despite being the same molecule. One explanation for this paradox lies in the duration of IL-6 exposure. Although chronic elevations are associated with insulin resistance, acute elevations may enhance insulin sensitivity (73). IL-6 also has regenerative, anti-inflammatory and anti-diabetogenic functions, when secreted as myokine by skeletal muscles during physical exercise. IL-6 released from skeletal muscle during exercise has been associated with improved glucose uptake and fat oxidation (74), suggesting that IL-6 has a crucial role in energy mobilization, even though IL-6 release from adipose tissue may not have the same effect (**Figure 2**).

Muscle contraction has been linked to an increase in plasma IL-6 levels, which is related to exercise intensity (75). IL-6 is one of the pathways through which physical activity improves insulin sensitivity, as it enhances GLUT-4 expression (76). Moreover, IL-6 can stimulate lipolysis and fat oxidation in humans without causing hypertriglyceridemia, which can have significant benefits for patients with metabolic syndrome (77). However, administering IL-6 exogenously as a mimetic for physical exercise is not an appropriate strategy, since elevated blood levels of IL-6 are still associated with inflammation (78), despite anti-inflammatory properties of muscle-derived IL-6.

The concentration of IL-6 has been inversely associated with 25-OH-D levels in older adults (79), suggesting that maintaining adequate 25-OH-D levels may reduce inflammation. A cohort study of over 5000 women and men found that n-3 PUFA intake was inversely correlated with plasma IL-6 levels (80). Two experimental studies examined the effect of whey protein or vitamin C and E supplementation on post-exercise inflammation. Both strategies were found to be useful in reducing plasma and muscle derived IL-6 levels, respectively, after moderate exercise (81, 82). Therefore, these results may be primarily due to a reduction in muscle rather than systemic inflammation.

IL-6 may present pro-inflammatory properties when derived from adipose tissue, but also anti-inflammatory effect derived from skeletal muscle. More studies are necessary to understand this paradoxal relationship of IL-6 and its impact on obesity management.

## System impact of physical exercise on inflammation

Physical exercise can be extremely helpful in managing inflammation and comorbidities associated with obesity. Exercise can significantly alleviate type II diabetes mellitus, as it promotes insulin sensitivity through various pathways, such as the reduction of plasma ceramides (83), enhancement of pancreatic β-cell function (84), and increased skeletal muscle capillarization (85). Exercise also improves lipid profile and cardiovascular health (86). Moreover, like adipose tissue, muscle tissue also acts as a secretory organ, releasing myokines to counteract inflammation and obesity-related comorbidities. Aerobic exercise can significantly decrease the level of inflammatory cytokines, when performed from moderate to high intensity with session lasting between 30 to 60 minutes in a frequency of 2 to 3 times per week (87).

Muscle-derived IL-6 can inhibit the production of inflammatory cytokines such as TNF-α and IL-1β, while promoting secretion of anti-inflammatory cytokines like interleukin-10 (IL-10) (88), thereby improving inflammatory profile in patients with sarcopenic obesity. The improved profile can attenuate age-related inflammation (inflammaging) and, consequently, improve glucose metabolism in skeletal muscle (89), as well as suppressing activation of macrophages, IL-2, and interferon-gamma (IFN-γ) (88).

Other myokine, such as irisin, are also associated with improvement of obesity-related conditions. Irisin is secreted by muscle and can induce white adipose tissue browning, which regulates energy metabolism (90). In addition, irisin has also been linked to beneficial effects on inflammation, neurodegeneration, oxidative stress, diabetes mellitus, and sarcopenia (91, 92). Strength training can play a role in activating mTOR and AMPK pathways for the secretion of these myokines (93). Moreover, physical exercise can also modify the composition of the gut microbiota that may improve systemic inflammation (94). Therefore, through several mechanisms, exercise can be used as an effective intervention to improve chronic inflammation in patients with obesity (95).

## Systemic impact of nutrition on inflammation

Dietary patterns are also essential for managing chronic low-grade inflammation in patients with obesity. It has been reported that high-fat diets, also described as Western diet, can be correlated with increased inflammatory profile and gut dysbiosis (96). These high-fat diets are mainly composed of ultra-processed foods, which appear to promote a chronic pro-inflammatory state (97), whilst diets with a higher content of whole, natural foods may reduce low-grade inflammation in obesity, placing particular emphasis on omega-3 polyunsaturated fatty acids, vitamins from vegetables and fruits (98), and dietary fiber (99). Providing prebiotics along with probiotics could also improve gut microbiota, reducing both systemic and intestinal inflammation (100). Other alternative supplements include branched-chain amino acids, calcium, and vitamin D3, which may reduce levels of TNF-α, IL-1β, and IL-6 (101).

The Mediterranean diet has been shown to improve several aspects for the management and prevention of obesity (102). The Mediterranean diet is characterized by increased consumption of fruits, vegetables, legumes, whole grains, olive oil, moderate intake of fish and dairy, and low intake of red meat. Moreover, the Mediterranean diet has been associated with improved biomarkers related to obesity comorbidities, such as glucose metabolism, plasma lipids, and cardiovascular diseases (103, 104). Therefore, following dietary patterns like the Mediterranean diet is currently recommended for reducing low-grade inflammation associated with obesity and sarcopenia (105, 106), as many of its components individually exhibit anti-inflammatory effects.

## Future perspective

This narrative review reinforces the view that obesity is a complex, multifactorial disease, characterized not only by excessive fat accumulation but also by chronic low-grade inflammation mediated by dysregulated cytokine secretion. Additionally, pro-inflammatory mediators such as IL-6, IL-1, and TNF-α contribute to the pathogenesis of obesity-related comorbidities including insulin resistance, cardiovascular disease, sarcopenia, and dyslipidemia.

Both physical exercise and evidence-based nutritional strategies can improve the inflammatory profile of patients with obesity, and consequently reduce the incidence of comorbidities. Exercise-induced myokines such as IL-6, IL-10, and irisin promote anti-inflammatory effects and improve metabolic outcomes. Moreover, certain dietary patterns, such as the Mediterranean diet and increased intake of nutrients with anti-inflammatory properties, including omega-3 fatty acids, polyphenols and antioxidants, can significantly reduce the secretion of pro-inflammatory cytokines and improve the metabolic profile of patients with obesity. Nevertheless, further research is needed to confirm the combined or isolated effect of physical exercise and nutrition on the management of obesity, especially longitudinal studies. Moreover, an important limitation of this review is the heterogeneity among studies, varying in outcomes assessed, intervention protocols, study design and duration.

In conclusion, addressing inflammation as a key component of obesity provides a more comprehensive approach to the treatment. Strategies combining regular physical exercise with appropriate nutritional interventions may offer a powerful tool to reduce inflammatory complications caused by obesity and obesity-related comorbidities. Investigations incorporating molecular biomarkers, microbiota profiling and individualized responses to interventions will be crucial for the development of precision therapies. Ultimately, treating inflammation as a core component of obesity may enhance current management strategies and improve long-term patient outcomes.
